# Abnormal Foot Position and Standing and Walking Ability in Rett Syndrome: an Exploratory Study

**DOI:** 10.1007/s10882-017-9585-6

**Published:** 2018-01-02

**Authors:** Hanneke E. Borst, Gillian S. Townend, Mirjam van Eck, Eric Smeets, Mariëlle van den Berg, Aleid Laan, Leopold M. G. Curfs

**Affiliations:** 1 Rett Expertise Centre Netherlands – Governor Kremers Centre, Maastricht University Medical Center, PO Box 616, 6200 MD Maastricht, The Netherlands; 2 Gemiva-SVG Groep, Gouda, The Netherlands; 3 HU University of Applied Sciences, Utrecht, The Netherlands; 4 Department of Rehabilitation Medicine, VU University Medical Center, Amsterdam, The Netherlands; 5 ICONE Orthopedics and Sports Traumatology, Schijndel, The Netherlands; 6 NRSV, Dutch Rett Syndrome Association, Utrecht, The Netherlands; 7 Reinaerde, Veenendaal Regio, Veenendaal, The Netherlands; 8 NVFVG, Dutch Association of Physiotherapy for People with Intellectual Disabilities, Amersfoort, The Netherlands

**Keywords:** Rett syndrome, Walking, Foot deformities, Orthosis, Weight bearing, Rett Syndrome Gross Motor Scale

## Abstract

This study aimed to determine whether there is a relationship between abnormal foot position and standing and walking ability in individuals with Rett syndrome (RTT), a rare neurological condition primarily affecting females, often accompanied by impaired gross motor function and musculoskeletal deformities. Through means of an online survey, physiotherapists were asked to share information about their work and experience with individuals with RTT. They were asked about their clients’ scores on the Rett Syndrome Gross Motor Scale and measures of their foot deformity, passive range of motion of dorsiflexion of the foot, use of supportive footwear, pressure load on the foot, and symmetry in weight bearing. 45 physiotherapists gave answers relating to 67 individuals with RTT who ranged in age from 2 to over 50 years. Almost 80% had an abnormal foot position which required support of special shoes or orthoses. Approximately 55% experienced abnormal pressure load on the foot and 65% demonstrated asymmetrical weight-bearing; 22% could sit independently and 17% were able to stand and walk independently. Of all the variables investigated, only abnormal distribution of pressure on the foot and asymmetry in weight bearing through the legs were found to be (negatively) correlated with standing and walking ability. Physiotherapists can use this information to give advice on othopedic support for the feet of individuals with RTT.

## Introduction

Rett syndrome (RTT) is a rare neurological disorder resulting mainly from a de novo mutation in the gene encoding methyl-CpG-binding protein 2 (*MECP2*) on the X-chromosome. It primarily affects females, with a prevalence of approximately 1 in 10,000 females (Leonard et al. 1997; Bienvenu et al. 2006; Laurvick et al. 2006; Fehr et al. 2011). The diagnosis of RTT remains primarily clinical in nature (Neul et al. 2010), with a stagnation in skills (often beginning between 6 and 18 months of age) after a period of seemingly normal early development, followed by a regression or loss of purposeful hand use and acquired spoken language and the appearance of characteristic stereotypic hand movements and gait abnormalities (Hagberg 2002; Monteiro et al. 2014). A stable period follows and, in some individuals, an improvement in skills can be achieved during this phase. However, autonomic dysfunction, respiratory irregularities, seizures, failure to thrive and abnormal muscle tone continue to be evident (Smeets et al. 2012), and general mobility seems to decline with age (Downs et al. 2008).

The gross motor skills of individuals with RTT are influenced by a range of neurological problems. The ability to walk, for example, is affected by abnormal muscle tone, ataxia, apraxia and disturbances in balance (Lotan and Hanks 2006; Downs et al. 2015). As an indicator of general walking ability within the Rett population, data extracted from the large-scale Australian Rett Syndrome Database (Downs et al. 2008) found that 30% of individuals were unable to walk compared with 43% who walked without support; the degree of support required was found to increase significantly with age (Downs et al. 2015). When considered in the context of early developmental milestones, it appears that those individuals who learn to stand and walk independently in the first two years of life are more likely to maintain the ability over a long period of time (Lotan and Hanks 2006). Yet the extent to which the post-regression deterioration in gross motor skills is due to the neurological issues mentioned above or due to lack of intervention is unclear. In certain cases, it has been shown that, following intensive physiotherapy intervention, individuals can learn to walk independently after many years of being unable to walk (Jacobsen et al. 2001; Larsson and Engerstrom 2001).

Musculoskeletal disorders also play a role in the acquisition and retention of gross motor skills. Early progressive scoliosis, peroneal hypotrophy and equinus (a deformation of the foot with limited dorsiflexion) are most frequently seen in individuals who have never been able to walk (Witt-Engerstrom and Hagberg 1990). Neuromuscular deterioration (for example, an increase of spasticity and /or dystonia) can contribute to scoliosis, hip dislocation, foot deformity, and contractures (Loder et al. 1989; Smeets et al. 2012), leading to a progressive loss of walking ability. Scoliosis is frequently seen in individuals with RTT (Kerr et al. 2003; Motil et al. 2008; Percy et al. 2010), and is positively correlated with delay, loss or absence of walking ability (Percy et al. 2010).

Sufficient mobility and stability of the foot and motor control are required in order to walk. Physical mobility is necessary to move the foot into the right position during the gait cycle and foot stability is essential for bearing the weight of the body and providing a stable base (Edington et al. 1990; Wernick and Volpe 1996). Decreased passive range of motion (PROM) in dorsiflexion of the ankle, as a consequence of shortening of the soleus and gastrocnemius muscles and the Achilles tendon, is reported in individuals with RTT (Smeets et al. 2012). This causes the foot to escape into an abnormal foot position such as equino-valgus (or occasionally equino-varus) position (Tahmasebi et al. 2015; Vulcano 2016). A flexible flatfoot in childhood may develop into a rigid flatfoot later in life, leading to valgus position of the calcaneus and shortening of the Achilles tendon, making walking difficult (Westhoff et al. 2011). Girls or women with abnormal foot position might benefit from the support that can be provided by shoe inserts or orthoses (Lotan and Hanks 2006).

The actual incidence of abnormal foot position and limitations in dorsiflexion in individuals with RTT is, however, unknown; so too is the precise relationship between these features and standing and walking ability. It is hypothesized by the authors that a correlation exists between the standing and walking ability of individuals with RTT and the combination of abnormal foot position, reduced mobility in the ankles and the need to support the feet.

Therefore, the main research question asked in the current study is: is there a relationship between abnormal foot position (e.g. equinus or flatfoot) and standing and walking ability in RTT?

Within this, the following sub-questions are examined:

- Is there a relationship between the passive range of motion (PROM) of dorsiflexion of the ankle and standing and walking ability?
- Is there a relationship between foot support, i.e. special footwear or orthoses, and standing and walking ability?
- Is there a relationship between an abnormal pressure load on the foot, when standing barefoot, and standing and walking ability?
- Is there a relationship between symmetry in weight-bearing of the legs, and standing and walking ability?

## Method

A cross-sectional design study was conducted, using an online survey to collect information on a cohort of girls and women with RTT. All had a clinical diagnosis of Rett syndrome, were living in the Netherlands and were being treated by a physiotherapist at the time of the study. Boys and men with RTT were excluded. No age limits were set.

### Data Collection

A questionnaire was developed, using Qualtrics (http://www.qualtrics.com/) as the online platform for hosting the survey, which was aimed at physiotherapists working with individuals with RTT. The therapists were invited by email to complete the survey online. In order to reach those physiotherapists most likely to be working with individuals with RTT, emails were distributed through the mailing lists of two specific Dutch physiotherapy organizations: the NVFVG (*n* = 70) and the Knowledge Network for Physiotherapists Working with Clients with Severe and Multiple Disabilities (a special interest group linked to the Association for the Care of the Disabled in the Netherlands (VGN) (*n* = 57). In addition, individual invitations were sent to physiotherapists whose names had been collected during an earlier survey for families conducted by the Rett Expertise Centre Netherlands (*n* = 16) (Townend et al. 2016).

The survey was divided into three sections: the first asked client-specific questions, the second about the physiotherapy delivered to these clients, and the final section focused on the therapists’ work experience and professional knowledge. The questions included open and closed (or multiple-choice) formats, with opportunities for free text responses. In the client-specific section, therapists were asked to answer a maximum of 35 questions for each individual with RTT with whom they were currently working. These questions asked about the client’s age, gross motor skills, musculoskeletal disorders, and specific features such as scoliosis and loss of walking ability. The therapists were expected to be able to answer based on their prior knowledge of their clients and from their case notes; they were not asked to assess their clients’ skills specifically in order to answer the survey. Age was assessed in six categories: 2–4 years, 4–12 years, 12–19 years, 19–40 years, 40–50 years and over 50. No identifying data was collected on the individuals with RTT.

This paper focuses on the questions relating to the individuals with RTT, and their standing and walking ability in particular.

A copy of the full questionnaire (in Dutch) can be requested from the authors.

### Rett Syndrome Gross Motor Scale

The main outcome variable discussed within this paper, the degree of support required for standing and walking, was scored using the question matrix from the Rett Syndrome Gross Motor Scale (RSGMS) which was incorporated into the online survey developed for this study. The RSGMS was adapted by Downs and colleagues (Downs et al. 2008, 2016) from the Gross Motor Function Measure (GMFM) specifically for use with individuals with RTT. It offers a 4-point ordinal scale for 15 items, describing the degree of support required for a range of gross motor skills, in which a score of 0 indicates the need for maximum support (or that the individual is unable to demonstrate that skill) and a score of 3 means that the individual can perform the skill independently. There are 3 sub-scales; Sitting, Standing and Walking, and Challenge. Together they give a maximum score of 45, which would indicate mastery of all assessed gross motor skills. The Sitting subscale includes three items: sitting on the floor, sitting on a chair (with backrest), and sitting on a stool (without backrest). Standing and Walking consists of nine elements: transfer from sitting to standing, standing for 3 s, 10 s and 20 s, walking, sidestepping, turning 180° degrees, walking up or down a slope, and stepping over an obstacle. The Challenge subscale includes: transfer from floor to standing, bending to pick up an object from the floor and return to standing, and running. The RSGMS is simple to complete and, as with the other questions in the survey, the respondents were expected to be able to answer from their usual case notes and their knowledge of their clients’ physical abilities. The reliability of the RSGMS is high (ICC 0.998) (Downs et al. 2016).

### Independent Variables

The first independent variable, the presence/absence of an abnormal foot position when standing barefoot, was scored dichotomously. Where an abnormal position was reported, this was scored nominally as a multiple choice (equinus, flexible flatfoot, rigid flatfoot or ‘other’). Support for the foot, for example (semi-)orthopedic shoes or ankle-foot orthosis (AFO), was likewise recorded as a nominal value. In addition, the therapists were asked to give the number of degrees of PROM of dorsiflexion of the ankle, as recorded by the Debrunner method. Finally, any abnormality in weight-bearing was scored as a nominal value for the feet (normal, almost normal, abnormal) and the legs (symmetrical, asymmetrical).

### Statistical Analysis

IBM Statistics SPSS 23 software was used to carry out the statistical analysis. The continuous variables (age and PROM) were assessed for normality. The remaining data were nominal or ordinal, which implies that these were not normally distributed. A Spearman correlation coefficient was used to investigate the relationship between the dependent and independent variables.

## Results

### Description of the Study Cohort

Seventy-one physiotherapists completed the questionnaires. However, 26 were excluded from this analysis because they did not answer the client-specific questions. The remaining 45 surveys gave information on 67 girls and women as several therapists reported on more than one client. The age of the cohort was normally distributed, with a range extending from under 4 (3%) to over 50 years of age (4.5%) (see Table 1).

**Table 1:** Description of study cohort (*N* = 67)

	Categories	Number (%)
Age range (years)	2–4	2 (3%)
	4–12	19 (28%)
	12–19	22 (33%)
	19–40	18 (27%)
	40–50	3 (4.5%)
	>50	3 (4.5%)
Type of abnormal foot position	Equinus	18 (27%)
	Flatfoot (flexible)	15 (22%)
	Flatfoot (rigid)	12 (18%)
	Other	7 (10.5%)
	No problems	10 (15%)
	Nil response	5 (7.5%)
Type of foot support	Semi-orthopedic footwear	14 (21%)
	Orthopedic footwear	17 (25.5%)
	Ankle-foot orthosis (AFO)	15 (22%)
	Other	4 (6%)
	Not required	10 (15%)
	Nil response	7 (10.5%)
Full foot on the floor (when standing with bare feet)	Yes	24 (36%)
	Partial	4 (6%)
	No	30 (45%)
	Nil response	9 (13%)
Symmetrical weight bearing through both legs (when standing)	Yes	12 (18%)
	No	46 (69%)
	Nil response	9 (13%)

### Foot Position and Use of Special Footwear

An abnormal foot position was reported in almost 80% of the cohort, with equinus (27%) and flexible flatfoot (22%) occurring most frequently (see Table 1). More than two-thirds had support from specialist footwear or an orthosis, of which orthopedic shoes (25.5%) were most common followed by AFOs (22%) and semi-orthopedic footwear (21%). When comparing between groups, orthopedic footwear and orthoses were most likely to be worn by clients with an equinus, and semi-orthopedic footwear by clients with a flexible flatfoot. The majority of clients wearing any type of special footwear were reported to have an abnormal foot position; only a minority of clients wearing special footwear were reported to have no abnormality. Almost half of the sample demonstrated an abnormal pressure load on the foot when barefoot, with the load mainly on their toes or heel or the lateral side of the foot. More than two-thirds (68%) were unable to weight bear symmetrically through both legs.

### Motor Skills

One-third of the cohort was able to sit independently on the floor, on a chair and on a stool. The majority required more support for the various standing and walking skills. Around 18% could stand and walk independently but less than 7% were able to demonstrate more complex skills such as sidestepping and turning through 180°. For further details, see Table 2.

**Table 2:** Levels of support for motor skills

	Maximum support	Medium support	Minimal support	Independent	Total
Sitting on the floor	**31 (48%)**	9 (14%)	4 (6%)	21 (32%)	65
Sitting on a chair	18 (27%)	20 (30%)	6 (9%)	**23 (34%)**	67
Sitting on a stool	**30 (47%)**	11 (17%)	9 (14%)	14 (22%)	64
Transfer from sitting to standing	**31 (51%)**	19 (31%)	2 (3%)	9 (15%)	61
Standing for 3 s	**25 (45%)**	14 (25%)	7 (12%)	10 (18%)	56
Standing for 10 s	**29 (52%)**	13 (23%)	4 (7%)	10 (18%)	56
Standing for 20 s	**31 (56%)**	10 (18%)	4 (7%)	10 (18%)	55
Walking 10 steps	**31 (58%)**	12 (23%)	1 (2%)	9 (17%)	53
Transfer from floor to standing	**46 (81%)**	6 (11%)	1 (2%)	3 (5%)	56
Sidestepping	**40 (73%)**	10 (18%)	3 (5%)	2 (4%)	55
Turning 180˚ degrees	**37 (69%)**	9 (17%)	4 (7%)	4 (7%)	54
Bending to pick up an object from the floor and return to standing	**42 (82%)**	6 (12%)	2 (4%)	1 (2%)	51
Stepping over an obstacle	**39 (75%)**	6 (11%)	5 (10%)	2 (4%)	52
Walk up or down a slope	**37 (70%)**	8 (15%)	6 (11%)	2 (4%)	53
Running	**50 (96%)**	1 (2%)	0 (0%)	1 (2%)	52

For each item, the level of support with the highest score is shown in bold

The average score in the Sitting subscale was 3.7, with a possible range from 0 to 9. For the Standing and Walking subscale, the average was 5.04 with a possible range of 0 to 27. The Challenge subscale had a lower average (0.54), in the context of a possible range from 0 to 9. The average total score was 8.68 (SD 11.3), within a possible range of 0 to 45. For further details, see Table 3.

**Table 3:** Motor skills scores

	Average score	SD	Range	N
PROM dorsiflexion (right)	0,35	16,8	*−70 / 30*	66
PROM dorsiflexion (left)	1,88	14,6	*−40 / 30*	66
Subscale Sitting	3,76	3,4	0–9	63
Subscale Standing and Walking	5,04	7,68	0–27	48
Subscale Challenge	0,54	1,56	0–9	50
Total RSGMS score	8,68	11,3	0–45	47

Normal PROM dorsiflexion in adults is 20 degrees. Responses ranged from -70 degrees dorsiflexion (indicating a great impairment in ankle motion) to 30 degrees dorsiflexion (normal range of motion)

### Correlations

Using the Spearman correlation co-efficient no relationship was found between PROM dorsiflexion left (r0.206, p0.161) or right (r0.193, p0.189) and Standing and Walking (see Table 4). Neither was there found to be a relationship between abnormal foot position and walking ability (r0.031, p0.833). There was, however, a weak but significant negative relationship between pressure load on the foot and the subscale Standing and Walking (r-0.470, p0001) and also between pressure load on the foot and walking ability (r-0422, p0.002) and total score on the RSGMS (R-0.507, p0.000). There was also a weak negative relationship between symmetry in weight bearing through both legs in standing (r-0.323, p0.031).

**Table 4:** Spearman’s rho for independent and dependent variables

	Item walking	Subscale Standing and Walking	Total score
Abnormal foot position	−0.078 (p 0.576)	−0.031 (p 0.833)	−0.024 (p 0.868)
Foot support	0.066 (p 0.646)	0.065 (p 0.663)	0.162 (p 0.282)
PROM left	−0.159 (p 0.250)	0.193 (p 0.179)	0.144 (p 0.4323)
PROM right	0.027 (p 0.846)	0.187 (p 0.194)	0.163 (p 0.264)
Pressure load on the bare foot when standing	−0.422 (p 0.002)*	−0.470 (p 0.001)*	−0.507 (p 0.000)*
Symmetrical weight bearing through both legs (when standing)	−0.225 (p 0.117)	−0.198 (p 0.188)	−0.323 (p 0.031)*

*p-value <0.05

**Fig. 1:**
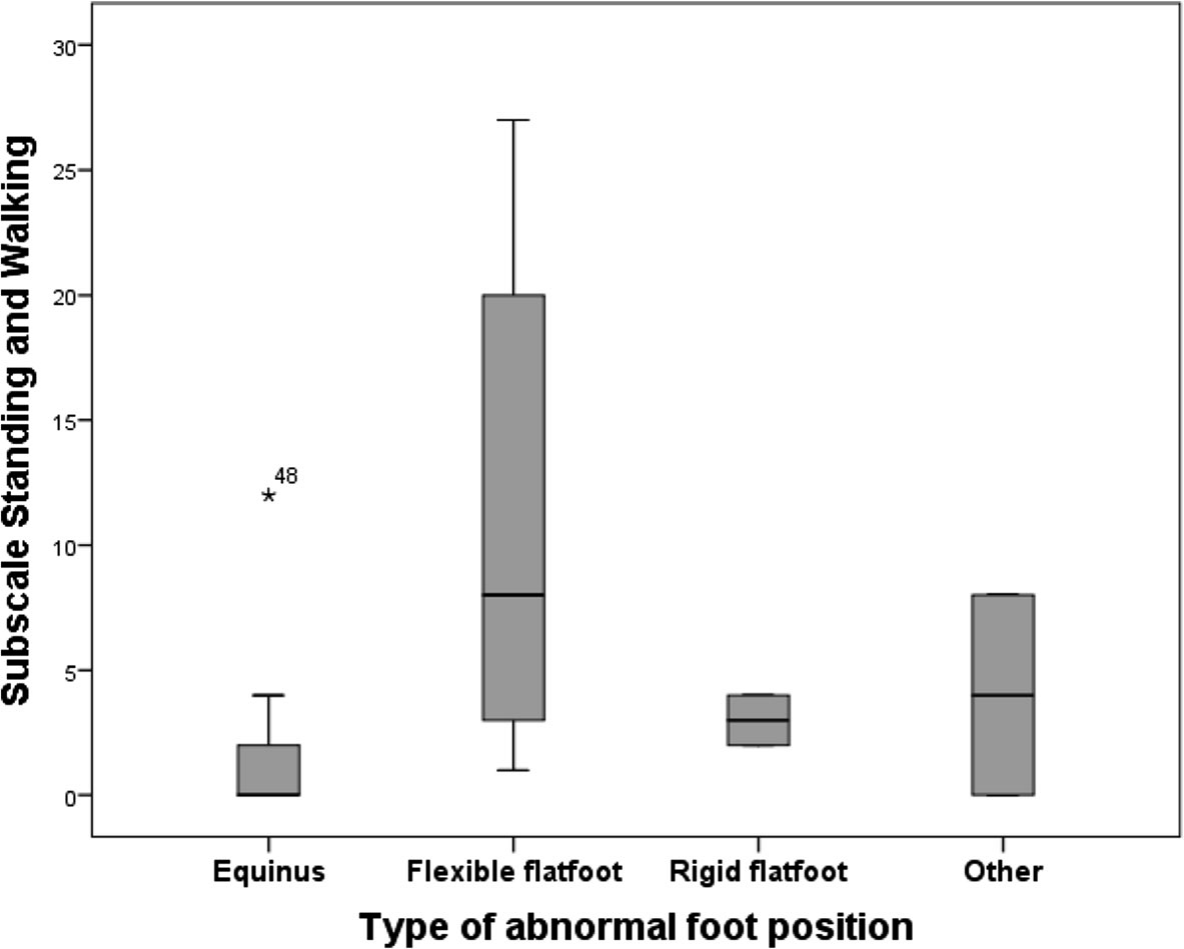
Average scores on the standing and walking subscale according to abnormal foot position

Further analysis of standing and walking ability according to different types of abnormal foot position revealed additional (non-significant) trends (see Fig. 1). For example, individuals with equinus, rigid flatfeet and other foot positions scored a low average on the Standing and Walking subscale (1,50 resp. 3,00 resp. 4,00) whilst those with flexible flatfeet achieved a higher average (10.70). This difference is greater than the Minimal Detectable Difference (MDD) of 4 points for this subscale, although not reaching statistical significance.

**Fig. 2:**
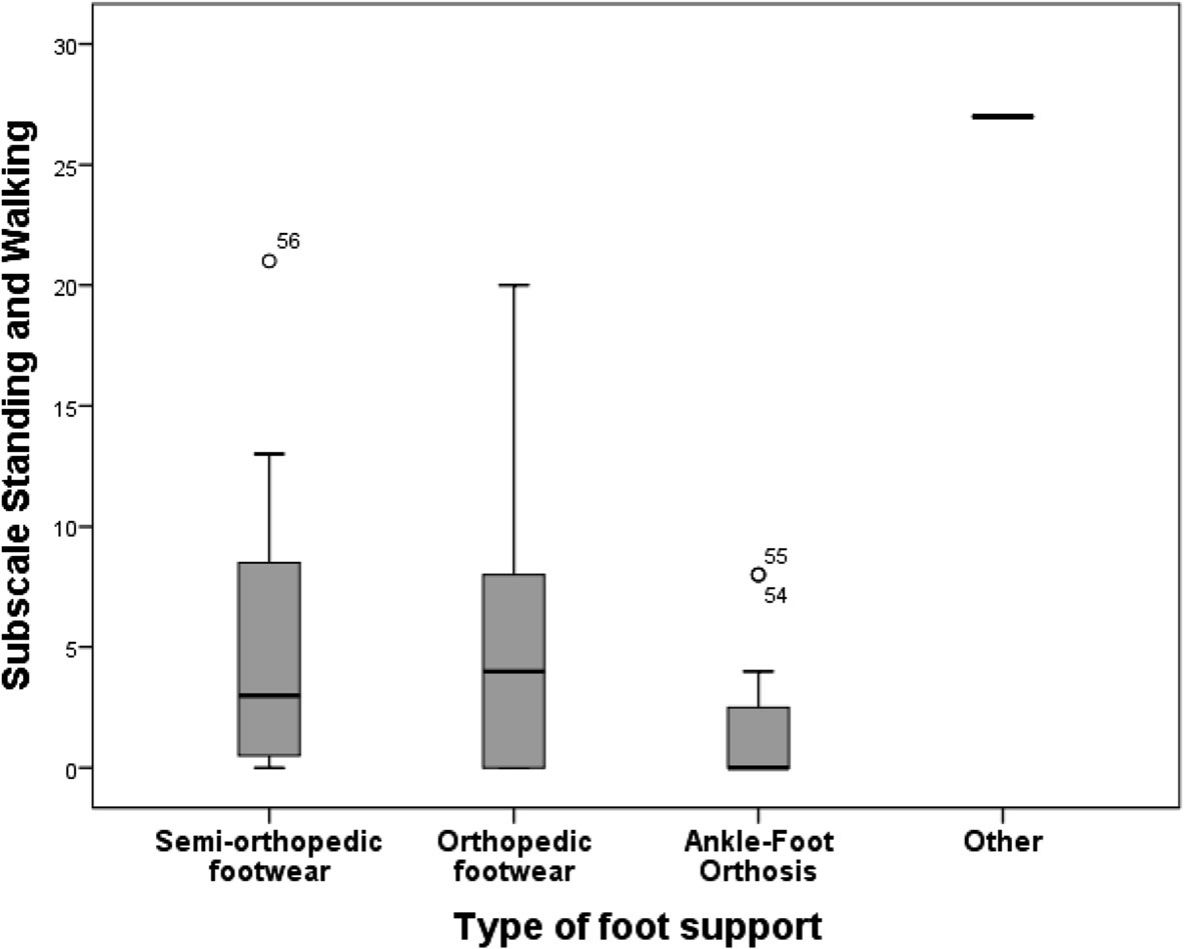
Average scores on the standing and walking subscale according to type of foot support

A weak trend was also evident in relation to the type of foot support and standing and walking ability. Individuals with AFOs averaged a score of 1.75 on the Standing and Walking subscale while those with semi-orthopedic or orthopedic footwear achieved a higher score (6.00 and 5.30 respectively) (see Fig. 2).

**Fig. 3:**
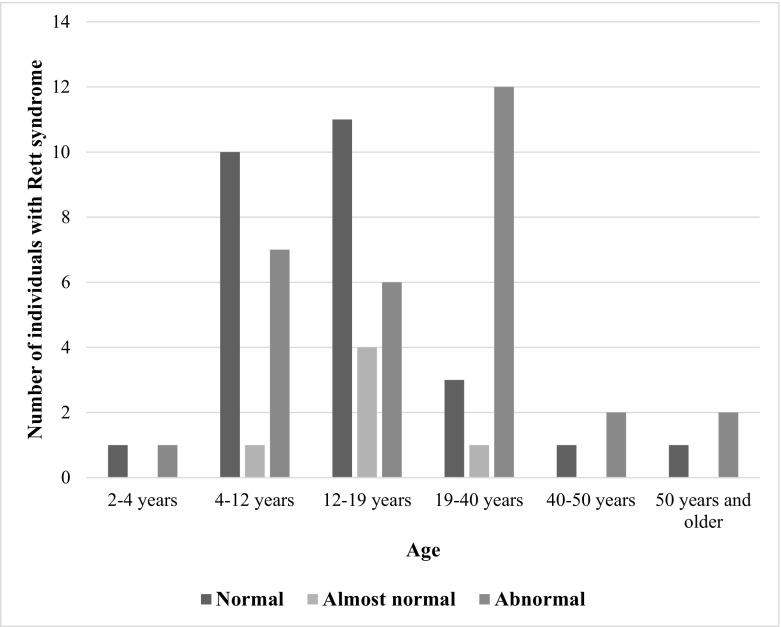
Frequencies of abnormal pressure load on the foot by age group

**Fig. 4:**
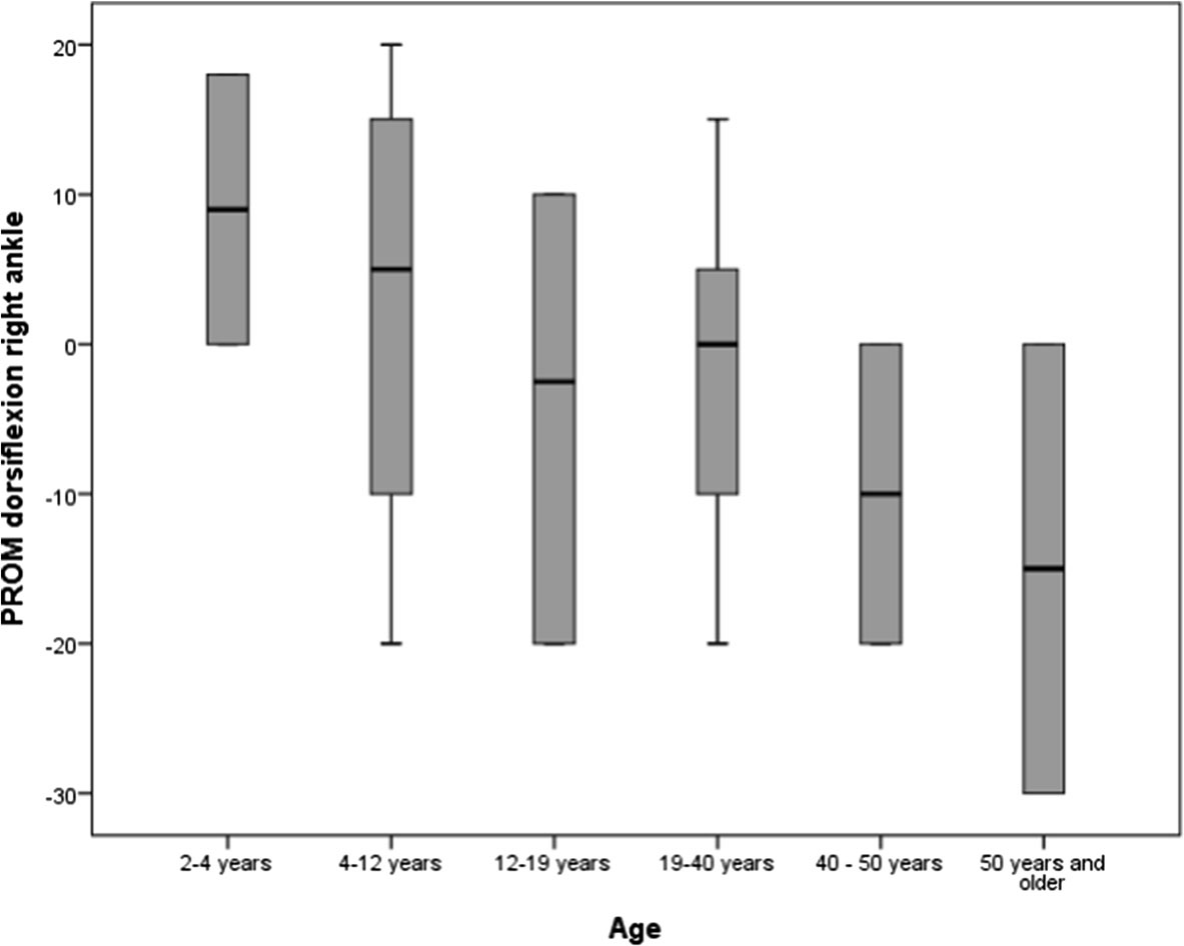
Relationship between age and PROM dorsiflexion of right ankle

**Fig. 5:**
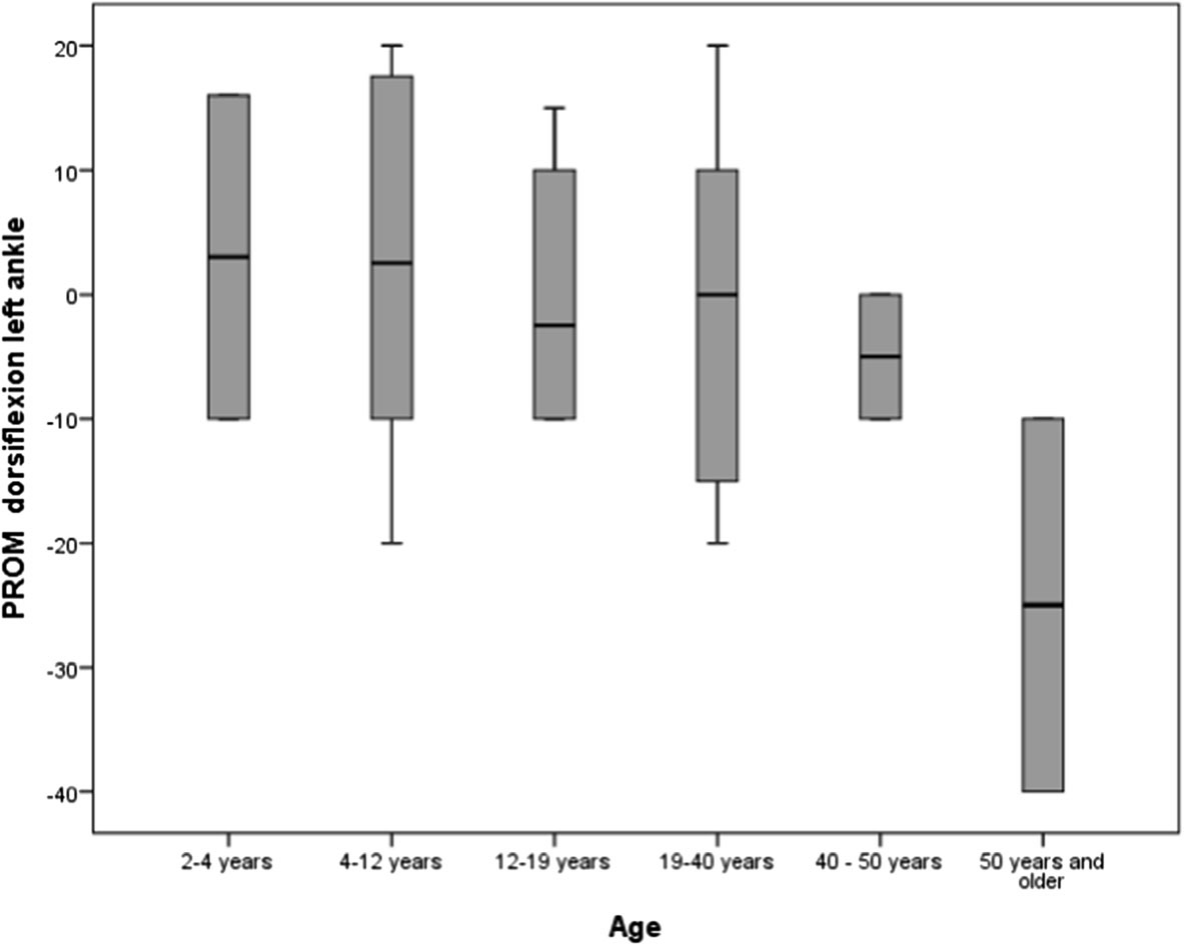
Relationship between age and PROM dorsiflexion of left ankle

Age was found to be positively correlated with distribution of pressure load on the feet while standing (r0.305, p0.016) (see Fig. 3). There was also a significant negative correlation between age and PROM of the right ankle (r-0.240, p0.050), which was not seen in the PROM of the left ankle (r-0.192, p0.119) (see Figs. 4 and 5), although the PROM left and PROM right were significantly correlated (r0.973, p0.000). The other variables did not show any correlation with age.

## Discussion and Conclusion

This study aimed to answer the question of whether abnormalities in foot position are related to standing and walking ability in individuals with RTT. We hypothesized that standing and walking ability would correlate with abnormalities in pressure load on the feet, asymmetry in weight bearing through the legs, use of supportive footwear and a smaller PROM of dorsiflexion of the ankle. Of all the variables investigated, only abnormal distribution of pressure on the foot and asymmetry in weight bearing through the legs were found to be negatively related with standing and walking ability.

The level of gross motor skills reported in this cohort was remarkably low in comparison with published literature. Other authors describe the prevalence of independent walking as between 43 and 70% (Cass et al. 2003; Downs et al. 2008, 2016), whereas in this study the prevalence was only 17%. Likewise, the percentages commonly reported by others (Cass et al. 2003; Downs et al. 2008) for independent sitting on a stool (70–90%) and standing from a chair (20–83%) were less evident in this population (22% and 15% respectively). It may be that there was a selection bias within the study population, as one of the inclusion criteria was ‘currently treated by a physiotherapist’. This could be interpreted as influencing the cohort in either of two ways: (1) those deemed to be most in need of treatment are those individuals with more severe physical limitations, therefore, fewer individuals with a higher level of motor skill may have been represented within the study cohort; or, (2) those in receipt of physiotherapy should (potentially) achieve higher skills because of the intervention.

Such a potential selection bias could be seen as a limitation of the study, also the fact that no specific instruction was given to the physiotherapists to assess their clients at the moment of completing the survey. Thus, accuracy of recall and recency of data on their clients’ levels of motor skills or foot position cannot be guaranteed. It was assumed, however, that as they were asked to report on clients being treated ‘currently,’ the information would be sufficiently up to date. The decision not to request contemporaneous assessment was deliberately taken in order to reduce the time burden on physiotherapists and thereby to encourage responses. A further limitation of this type of survey is that it was not possible to determine causality between the independent variables. This would be an interesting area for further research.

In addition, potential confounders should be noted. These include levels of activity and type and frequency of physiotherapy intervention, genetic mutation, and age. Questions were not asked about how many hours of exercise an individual engaged in per day or week nor the type of physiotherapy regime they underwent. In this respect it was not possible to determine the effects of any physiotherapy interventions beyond the provision of special footwear. Mutation types were not explored as physiotherapists in the Netherlands do not usually have access to this information. Age was considered. It is often reported that gross motor skills are negatively associated with age (Downs et al. 2008) and, based on the literature (Cass et al. 2003; Downs et al. 2008, 2016), it was expected that standing and walking ability would be found to decrease with age in the study. Whether this might represent a true decline or be the result of the reduced physiotherapy input individuals often experience after the age of 18 was felt to be open to question. However, the anticipated decline was not confirmed. No significant relationship was found between age and any of the subscales nor with the total score on the RSGMS. This may suggest that physiotherapy intervention can ameliorate a decline in motor abilities, but, on its own, this evidence is not strong enough. As any decline is generally noted to be very slow, longitudinal studies which shed more light on the issue would be welcomed in the future. Examining the effect of physiotherapeutic interventions on walking ability, and specifically on motor deterioration in the older age group, could potentially offer new insights to challenge the current assumption of motor decline in the late stage of Rett syndrome.

One aspect where age was found to have a positive correlation was distribution of pressure load on the feet while standing. There was also a significant negative correlation between age and PROM of the right, but not of the left, ankle. Despite the fact that no other variables showed a correlation with age in this cohort, it is known that musculoskeletal disorders such as scoliosis worsen with age (Lotan and Hanks 2006; Downs et al. 2009) and it is hypothesized these impact both distribution of pressure load on the feet and standing and walking ability. In this study, an abnormal distribution of pressure on the foot did appear to have a negative relationship with standing and walking skills. Although there was no significant correlation between the use of special footwear and standing and walking ability, clients with orthoses seemed to have lower scores on the Standing and Walking subscale of the RSGMS. This could be related to the lower scores in clients with equinus and rigid flatfoot, as most clients with those foot positions were also reported to wear orthoses. An AFO reduces ankle mobility which will in turn influence (potential) walking ability. Physiotherapists should consider this when advising parents on the pros and cons of special footwear.

Supporting the feet through the use of appropriate footwear and/or orthoses could help to improve function by compensating for an abnormal axis, thereby enabling more effective muscle tone, as well as correcting foot position which contributes to a better walking pattern. Good monitoring of the foot position and muscle length and tone is, therefore, of great importance. As we found a trend towards clients with equinus and rigid flatfoot having lower standing and walking ability, it may be that interventions which aim to maintain as normal a foot position as possible could help to maintain motor function. Lotan and Hanks (2006) advise preventive intervention when addressing fixed deformities in order to prevent further loss of standing and walking ability. Interventions may include orthoses, regular use of a standing frame, serial casting, botulinum toxin injections or surgery. Ankle-foot orthoses, botulinum toxin and surgery have been proven to be effective in improving gait function in individuals with cerebral palsy who have foot deformities (Ries et al. 2015; Park et al. 2006; Choi et al. 2016) and may prove equally helpful for individuals with Rett syndrome. It is important that physiotherapists advise parents and caregivers on how best to support the feet, in order to maintain normal foot position and motor function and to prevent a flexible flatfoot becoming a rigid flatfoot, for as long as possible. Once rigid, the options for treatment are narrower. Any functional limitations imposed by support or orthoses, therefore, should be viewed in the context of preventing or delaying the need for more major, bony surgery. When supporting the feet is not sufficient to maintain standing and walking ability, however, an orthopedic surgeon should be consulted, and the pros and cons of the possible interventions need to be considered.

This study is the first time that the RSGMS has been used for research purposes in the Netherlands. One possible disadvantage of recruiting through physiotherapists, and asking only about the clients they were currently treating, is that data was not collected on all individuals with RTT in the Netherlands. Nevertheless, this study demonstrated that the RSGMS is a simple-to-complete clinical instrument that is easy to apply in practice. The subscales are useful in distinguishing between different sub-skills and in demonstrating the influence of different disorders on various gross motor skills. The study offers a picture of the gross motor levels of girls and women with RTT who are being treated by a diverse group of physiotherapists the Netherlands.

As a result of the findings, further research into the relationship between abnormal foot position and gross motor skills is recommended. Longitudinal studies of musculoskeletal disorders and gross motor skills can provide more insight into the development of these disorders and the limitations that they place on motor skills. In addition, more detailed analysis of walking and other motor skills can contribute to a more precise understanding of the relationship between an abnormal foot position and walking. The relationships between frequent musculoskeletal disorders such as scoliosis, subluxations of the hip and contractures of the lower extremities, as well as stereotypies and muscle tone related to standing and walking ability, are also worth investigating, as are the effects of physical interventions on long-term motor function, something currently thought to decline with age. Finally, it is recommended that further research be conducted into physiotherapy interventions that aim to develop better foot and ankle function.
